# A review of the psychometric properties of the Health of the Nation Outcome Scales (HoNOS) family of measures

**DOI:** 10.1186/1477-7525-3-76

**Published:** 2005-11-28

**Authors:** Jane E Pirkis, Philip M Burgess, Pia K Kirk, Sarity Dodson, Tim J Coombs, Michelle K Williamson

**Affiliations:** 1 School of Population Health, The University of Melbourne, Melbourne, Australia; 2 School of Population Health, The University of Queensland, Brisbane, Australia; 3 New South Wales Institute of Psychiatry, Sydney, Australia

**Keywords:** Mental health, outcome measurement, Health of the Nation Outcome Scales (HoNOS), Health of the Nation Outcome Scales for Children and Adolescents (HoNOSCA), Health of the Nation Outcome Scales 65+ (HoNOS65+)

## Abstract

**Background:**

The Health of the Nation Outcome Scales was developed to routinely measure outcomes for adults with mental illness. Comparable instruments were also developed for children and adolescents (the Health of the Nation Outcome Scales for Children and Adolescents) and older people (the Health of the Nation Outcome Scales 65+). All three are being widely used as outcome measures in the United Kingdom, Australia and New Zealand. There is, however, no comprehensive review of these instruments. This paper fills this gap by reviewing the psychometric properties of each.

**Method:**

Articles and reports relating to the instruments were retrieved, and their findings synthesised to assess the instruments' validity (content, construct, concurrent, predictive), reliability (test-retest, inter-rater), sensitivity to change, and feasibility/utility.

**Results:**

Mostly, the instruments perform adequately or better on most dimensions, although some of their psychometric properties warrant closer examination.

**Conclusion:**

Collectively, the Health of the Nation Outcome Scales family of measures can assess outcomes for different groups on a range of mental health-related constructs, and can be regarded as appropriate for routinely monitoring outcomes.

## 

The Health of the Nation Outcome Scales (HoNOS) arose out of the UK's Health of the Nation Strategy, and was created by Wing and colleagues as an instrument that could be routinely used to measure outcomes for adults with mental illness [1,2]. Comparable measures for children and adolescents (HoNOSCA) and older people (HoNOS65+) were later developed by Gowers and colleagues [3,4] and Burns et al [5], respectively.

All three instruments measure mental health and social/behavioural functioning (see Table 1), and are being used increasingly as routine clinical outcome measures against which the quality and effectiveness of mental health services can be monitored, judged and improved. They are the most widely used routine outcome measures in British mental health services [6], and they are being used at admission, review and discharge in inpatient and ambulatory public-sector mental health services in all Australian states/territories [7]. They are also being used widely in New Zealand, and, to a greater or lesser degree, in other countries, including Canada, Denmark, France, Italy, Germany and Norway.

**Table 1:** Items, structure and scoring for the HoNOS family of measures

**Instrument**	**Item**	**Subscales/sections**	**Scoring**
**HoNOS**	1. Overactive, aggressive, disruptive or agitated behaviour 2. Non-accidental self-injury 3. Problem drinking or drug taking 4. Cognitive problems 5. Physical illness or disability problems 6. Problems associated with hallucinations and delusions 7. Problems with depressed mood 8. Other mental and behavioural problems 9. Problems with relationships 10. Problems with activities of daily living 11. Problems with living conditions 12. Problems with occupation and activities	Behaviour (1–3) Impairment (4–5) Symptoms (6–8) Social (9–12)	Each item rated on a 5-point scale: 0. no problem 1. minor problem requiring no action 2. mild problem but definitely present 3. moderately severe problem 4. severe to very severe problem. Scoring yields individual item scores, subscale scores and a total score.

**HoNOSCA**	1. Problems with disruptive, antisocial or aggressive behaviour 2. Problems with over-activity, attention or concentration 3. Non-accidental self-injury 4. Problems with alcohol, substance or solvent misuse 5. Problems with scholastic or language skills 6. Physical illness or disability problems 7. Problems associated with hallucinations, delusions or abnormal perceptions 8. Problems with non-organic somatic symptoms 9. Problems with emotional and related symptoms 10. Problems with peer relationships 11. Problems with self-care and independence 12. Problems with family life and relationships 13. Poor school attendance 14. Problems with knowledge or understanding about the nature of the child or adolescent's difficulties 15. Problems with lack of information about services or management of the child or adolescent's difficulties	Section A (1–13) Behaviour (1–4) Impairment (5–6) Symptoms (7–9) Social (10–13) Section B (14–15)	Each item rated on a 5-point scale: 0. no problem 1. minor problem requiring no action 2. mild problem but definitely present 3. moderately severe problem 4. severe to very severe problem. Scoring yields individual item scores, subscale scores and a total score (derived from Section A only).

**HoNOS65+**	1. Behavioural disturbance (e.g., overactive, aggressive, disruptive or agitated behaviour, uncooperative or resistive behaviour); 2. Non-accidental self-injury; 3. Problem drinking or drug taking; 4. Cognitive problems; 5. Physical illness or disability problems; 6. Problems associated with hallucinations and delusions; 7. Problems with depressive symptoms; 8. Other mental and behavioural problems; 9. Problems with relationships; 10. Problems with activities of daily living; 11. Problems with living conditions; and 12. Problems with occupation and activities	Behaviour (1–3) Impairment (4–5) Symptoms (6–8) Social (9–12)	Each item rated on a 5-point scale: 0. no problem 1. minor problem requiring no action 2. mild problem but definitely present 3. moderately severe problem 4. severe to very severe problem. Scoring yields individual item scores, subscale scores and a total score.

Despite their relative widespread use as outcome measures, there is some reported concern – particularly among clinicians who are using the instruments. Anecdotally, some clinicians question the psychometric soundness of the instruments, and argue that they do not have good clinical utility [7]. With the exception of a specific review of the applicability of the HoNOS and the HoNOS65+ for older people [8], there has been no comprehensive review of these instruments that can inform this debate. The current paper fills this gap, by appraising the psychometric properties of each.

## Methods

The review could best be described as a qualitative systematic review [9]. It involved a comprehensive search of all potentially relevant articles, using explicit search criteria. However, because it assessed the psychometric properties of three different instruments on eight different dimensions, it was beyond its scope to statistically combine the results of different studies. Instead, the results were summarised in a narrative fashion.

### Article retrieval

Searches of the electronic databases MEDLINE and PSYCINFO were conducted from their respective years of inception to November 2005. The search was retrieved articles using the following search terms:

• MENTAL HEALTH or PSYCHIATR*

• OUTCOME MEASURE* or ROUTINE OUTCOME MEASURE*;

• HEALTH OF THE NATION OUTCOME SCALES or HONOS;

• HEALTH OF THE NATION OUTCOME SCALES 65+ or HONOS65+; and

• HEALTH OF THE NATION OUTCOME SCALES FOR CHILDREN AND ADOLESCENTS or HONOSCA.

Potentially relevant peer-reviewed journal articles were retrieved by this means, and their reference lists scanned for further pertinent articles. Efforts were also made to retrieve government and other reports, both from within Australia and overseas, largely by conducting Internet searches using the above terms. Greatest weight was given to the peer-reviewed articles for two reasons. Firstly, it was possible to be confident that they had undergone some academic checking for scientific merit. Secondly, this approach created a relatively 'level playing field' for all instruments. It is acknowledged, however, that the relative standing of the given journal was not taken into account, and the individual studies were not systematically rated for quality (although consideration was given to the strength of their design).

In addition, the review primarily concerned itself with articles (and reports) that involved explicit testing of the psychometric properties of a given instrument (e.g., a study that examined the validity and reliability of the HoNOS). Articles that described the use of a given instrument in a study of some other kind (e.g., a randomised controlled trial that used the HoNOS as an outcome measure in assessing the relative merits of two different types of treatment) were given less weight. This decision was made on the grounds that the latter type of study, by design, implicitly accepted the psychometric value of the given instrument and to use the findings as evidence for the psychometric robustness of that instrument would create a somewhat circular argument.

### Critical appraisal of the instruments

Evidence from the above articles and reports was used to critically appraise each of the instruments. The critical appraisal exercise was guided by a checklist that drew on the work of Greenhalgh et al [10], Green and Gracely [11], McDowell and Newell [12] and Chronbach and Meehl [13].

Specifically, the checklist elicited evaluative information on each instrument, namely its:

• Content validity, which refers to the instrument's comprehensiveness (i.e., how adequately the sampling of items reflects its aims), and is commonly ascertained by asking stakeholders to review the content of the instrument;

• Construct validity, which involves conceptually defining the construct to be measured by the instrument, and assessing the internal structure of its components and the theoretical relationship of its item and subscale scores;

• Concurrent validity, which pits the instrument against 'gold standards' (e.g., scores on more established instruments);

• Predictive validity, which assesses the instrument's ability to predict future outcomes (e.g., resource use or treatment response);

• Test-retest reliability, or the degree of agreement when the same instrument is applied to the same consumer by the same rater at two different time points;

• Inter-rater reliability, or the degree of agreement when the same instrument is applied to the same consumer by different raters at the same time point;

• Sensitivity to change, or the degree to which the instrument demonstrates change over time, as measured against 'gold standards' (e.g., change assessed by more established instruments); and

• Feasibility/utility, or the degree to which the instrument is acceptable to and useful for stakeholders.

## Results

### HoNOS

#### Content validity

Shergill et al [14], Orrell et al [15] and McClelland et al [16] explored the content validity of the HoNOS by asking consumer/carer advocacy groups and mental health professionals to comment on whether its items reflected areas of concern for them. In the main, respondents in these studies were positive, suggesting that the HoNOS was appropriate, well-designed and thorough.

However, respondents were concerned about the restriction imposed by the rater being forced to indicate only one problem in Item 8 (Other mental and behavioural problems) [14,16], and questioned the ability of Item 6 (Problems associated with hallucinations and delusions) to accurately describe the symptoms and role performance of a person with schizophrenia [15]. They also felt that the social items (Items 10, 11 and 12) were problematic because the complexity of information needed to rate them [15,16].

Respondents also noted that, for some items, anchor points and their associated terminology were subjective [14,15]. They commented on difficulties with knowing which item to use for rating some symptoms, such as elated mood. In addition, they observed the failure of the instrument to take into account factors such as culture, poverty, abuse, safety and risk, bereavement and medication compliance [14,15]. Some respondents suggested that the HoNOS was open to human error and misinterpretation [16].

#### Construct validity

In studies of the internal consistency of the HoNOS, Cronbach's alpha has ranged from 0.59 to 0.76, indicating moderately high internal consistency and low item redundancy, and supporting the instrument's use as a meaningful summary of severity of symptoms [1,14-20]. That said, Trauer [18,21] has argued that the HoNOS does not measure a single, underlying construct of mental health status.

McClelland et al [16] examined the relative contribution of each of the HoNOS items to the total score, and found that Item 7 (Problems with depressed mood), Item 8 (Other mental and behavioural problems) and Item 9 (Problems with relationships) had the greatest weight, contributing 15%, 19% and 14% to the total, respectively. By contrast, Item 11 (Problems with living conditions) and Item 12 (Problems with occupation and activities) contributed only 3% each.

Preston [22], Trauer [18] and McClelland [16] examined the subscale structure of the HoNOS. In his study, Preston found that the four factor model defined by the original subscales had good fit, but that the contribution of individual items to their respective subscales varied in two separate mental health services, indicating differentiation in construct interpretation. Trauer's examination of the subscales revealed a poorer fit than Preston's, leading him to propose an alternative five factor structure which has been supported in later studies [20]. McClelland's study also identified alternative factors.

#### Concurrent validity

Numerous studies have considered the concurrent validity of the HoNOS, assessing its performance against more established instruments that have been shown to validly measure related constructs. In the main, the HoNOS has been shown to perform well against clinician-rated instrument such as the Role Functioning Scale [1], Brief Psychiatric Rating Scale [1,14-16], Global Assessment Scale [14-16,23-25], Life Skills Profile [20,23], Manchester Audit Tool [26], Clifton Assessment Procedures for the Elderly – Behaviour Rating Scale [14], Clinical Dementia Rating [14], Mini-Mental State Examination [14], Schedules for Clinical Assessment in Neuropsychiatry [25,27], Broad Rating Schedule [25], Disability Assessment Schedule [25], Social Adjustment Scale [25], Location of Community Support Scale [15], Social Behaviour Schedule [15,27], Hamilton Rating Scale for Depression [28] and Positive and Negative Symptoms Scale [28]. There are some exceptions, with low correlations being found between the HoNOS and the Brief Psychiatric Rating Scale in one study [29] and the Beck Depression Inventory in another [19].

By contrast, the HoNOS has shown poor or mixed performance against consumer-rated instruments such as the Symptom Check List 90 – Revised [29,30], Social Adjustment Scale [29], Medical Outcomes Study Short Form 36 [30], Camberwell Assessment of Need Short Appraisal Schedule [31], Quality of Life Scale [14], Avon Mental Health Measure [32], Outcome of Problems of Users of Services [32], an instrument adapted from the Quality of Life Index for Mental Health [23] and even a self-rating version of the HoNOS with a similar question structure [33]. As with the clinician-rated measures, there are exceptions to the general rule, but even where studies have reported correlations between the HoNOS and consumer-rated measures – e.g., the Camberwell Assessment of Need Short Appraisal Schedule [34-36], Medical Outcomes Study Short Form 36 [15,28], General Health Questionnaire [15] and Comprehensive Quality of Life Scale [28] – they tend to vary across domains and be lower than those between the HoNOS and clinician-rated measures. These findings are not surprising, given that poorer correspondence is typically found between instruments that rely on information from informants of different classes than those which rely on information from informants of the same class, since different informants have access to different information.

The ability of the HoNOS to discriminate between consumer groups differentiated on a range of treatment- and service-based indicators has also been used to test its concurrent validity. Several studies have found high total scores on the HoNOS to be associated with diagnoses of drug and alcohol, psychotic and bipolar disorders, high scores on items relating to hallucinations/delusions and social and cognitive problems to be associated with a diagnosis of schizophrenia, high scores on items relating to aggressive behaviour, drinking/drug taking and anxiety to be associated with a diagnosis of mania, and high scores on items relating to suicidal thoughts/behaviours, physical illness and depressed mood to be associated with a diagnosis of depression [16,20,24,26,37,38]. Similarly, a number of studies have found that the HoNOS can discriminate between consumers with differing levels of need or disability, as indicated by their current or expected location of treatment – e.g., those receiving standard case management versus those assertive case management [39], those in residential/nursing home, day patient, outpatient and inpatient settings [14,15,28], and those in long-stay settings with low, medium and high expectations of discharge [40].

#### Predictive validity

Several studies have examined the predictive validity of the HoNOS. Most have found it to have reasonably good predictive validity, explaining a significant proportion of the variance in resource use (e.g., as measured by service contacts, length of stay and costs) and treatment outcome (e.g., as measured by readmission rates, retention in the community, treatment response and death) [23,28,41-43]. There have been exceptions, however, with some studies finding limited correspondence between HoNOS total scores and resource use [44,45].

#### Test-retest reliability

Few studies have examined the test-retest reliability of the HoNOS, but those that have generally report fair to moderate overall reliability scores [14,15,30]. Particularly low reliability scores have been reported for Item 1 (Overactive, aggressive, disruptive or agitated behaviour), Item 3 (Problem drinking or drug taking), Item 7 (Problems with depressed mood), and Item 10 (Problems with activities of daily living).

#### Inter-rater reliability

Most studies of the inter-rater reliability of the HoNOS total score have found that the overall agreement between pairs of raters is fair to moderate [14,27,30], or even moderate to good [1,15,25,28], but that agreement is poor on particular items. Items identified as problematic include Item 4 (Cognitive problems) [27], Item 7 (Problems with depressed mood) [27], Item 8 (Other mental and behavioural problems) [1,27], Item 9 (Problems with relationships) [15], Item 11 (Problems with living conditions) [15,46] and Item 12 (Problems with occupation and activities) [1,27,46].

#### Sensitivity to change

The sensitivity of the HoNOS to change has been assessed in a number of studies which have examined the extent to which the direction and magnitude of movement in HoNOS total or item scores correlates with some external measure of change.

The simplest of these studies have examined change in HoNOS over time in given settings, hypothesising that there should be a decrease in severity as the consumer nears the end of an episode. Generally, these studies have found decreases of the greatest magnitude in inpatient settings and of lesser magnitude in community settings [16,46-48]. That said, there is some evidence that there may be an interaction between setting, diagnosis and severity, and that the HoNOS may be able to detect change in the community for those with depression and anxiety [26] and those with higher HoNOS total scores at episode start [49]. Particular items may also interact with setting, with one study that considered the range of inpatient and community settings finding that scores on all items except Item 11 (Problems with living conditions) showed decreases over time [16], and another that concentrated on a community setting only finding that only Items 7 (Problems with depressed mood), 8 (Other mental and behavioural problems) and 9 (Problems with relationships) had sufficient relevance and variability to change over time [48].

Other studies have used clinician or consumer judgement as the 'gold standard' against which to evaluate whether change has occurred and, if so, whether the HoNOS is capable of detecting it. In separate studies, Taylor and Wilkinson [50] and Gallagher and Teesson [39] found correlations between changes in consumers' HoNOS total scores and clinical judgements about whether they had improved, remained stable or deteriorated made by GPs and case managers, respectively. Likewise, Hunter et al [32] found that significant decreases in HoNOS total scores between initial and repeat ratings corresponded with consumers' self report of their goals having been met.

Still other studies have compared the HoNOS's dynamic properties and capacity to detect change against other, more established measures of outcome. Using these criteria, McClelland et al [16] found the HoNOS to perform commensurately with the Global Assessment Scale and the Brief Psychiatric Rating Scale. Sharma et al [51] found it performed well against the Modified Clinical Global Impressions Scale, although the correlations were greatest for those with extreme improvement or deterioration. Ashaye et al [43] found the HoNOS was correlated with the Clifton Assessment of Strengths, Interests and Goals and two quality of life scales in elderly consumers, particularly those with dementia and depression. By contrast, Bebbington et al [27] found the HoNOS performed poorly by comparison with the Schedules for Clinical Assessment in Neuropsychiatry and the Social Behaviour Schedule.

A final approach to examining sensitivity to change has involved assessing whether improvements in HoNOS total scores are observed for consumers who receive evidence-based therapies and therefore would be expected to show reductions in symptom severity. Bech et al [37], for example, hypothesised that consumers who received lithium and/or ECT would show greater improvement on the HoNOS than consumers who did not, and found this to be the case, at least for the Behaviour and Symptoms subscales.

#### Feasibility/utility

There has been considerable debate about the feasibility/utility of the HoNOS. The least enthusiastic authors have argued that it is of limited value in informing care planning [24,51-55]. More positive authors have suggested it is a comprehensive, user-friendly tool that is likely to have utility in routine outcome measurement [1,16,19,28,38,39,56], and, with other evidence, could make a valuable contribution in informing clinical judgements [2].

Audits of the extent to which the HoNOS is being used in particular settings have generally lent support to the latter view. Glover and Sinclair-Smith [57] found that 60% of mental health care provider trusts in Britain had implemented routine outcome measurement (with the majority using the HoNOS), and James and Kehoe [58] found that 77% of consumers in a UK district service had HoNOS scores recorded in their care plans. The latter finding was supported by Broadbent [41], who found that the HoNOS was completed for the majority of consumers on an electronic case register in the UK. In a trial in New Zealand, Eagar, Trauer and Mellsop [20] found that 95% of episodes of care had at least one HoNOS completed (and that the majority had few missing items), although only 58% had one completed at the beginning and the end of the episode.

Reports of clinicians' experiences with using the HoNOS have been more mixed. James and Kehoe [58], Broadbent [41] and Milne et al [59] found that UK clinicians were relatively positive about the HoNOS, viewing it as potentially useful, but insisting that its ongoing use would depend on adequate resourcing, infrastructure, training and feeback. By contrast, Gilbody [54] found that many UK psychiatrists questioned the instrument's usefulness. In field trials conducted in Australia, Trauer [60] found that clinicians at one site were extremely positive about the HoNOS, whereas those at four others were more ambivalent, believing that it contributed only minimally to their treatment practices.

### HoNOSCA

#### Content validity

No studies available.

#### Construct validity

Gowers et al [3,4] and Harnett et al [61] examined the internal structure of the HoNOSCA during its development, considering both individual items and subscales. They considered the correlations between the individual items and found them to be low, which they took as evidence that each item carried independent weight. They then examined the factor structure of the HoNOSCA, and found that it generally mirrored the instrument's subscales. Brann [62], by contrast, also examined the factor structure of the HoNOSCA and produced preliminary evidence for a different set of factors. Neither Gowers et al nor Brann found support for the instrument's sections.

Gowers et al [3,4] also considered the extent to which the HoNOSCA total score accurately reflected clinical severity, arguing that high total scores should more frequently be associated with high scores on a few items than on mild to moderate scores on a number of items. They found that the total score increased as a linear function of high individual item scores, a finding confirmed by Brann et al [63] in a subsequent study.

#### Concurrent validity

Several studies have weighed up the HoNOSCA's performance against other measures. Studies that have examined the correlation between the HoNOSCA total score and scores on other clinician-rated measures have typically reported moderate correlations (r = 0.6 or above). This was the case when the HoNOSCA was compared with the Children's Global Assessment Scale [64], the Paddington Complexity Scale [61,64], and the Global Assessment of Psychosocial Disability [65].

Studies that have evaluated the HoNOSCA against parent- and child/adolescent-rated instruments have typically produced lower correlations. Yates et al [64] found only modest correlations between the HoNOSCA and the Behaviour Check List, Strengths and Difficulties Questionnaire, Child Health Related Quality of Life Questionnaires and Modified Harter Self-Esteem Questionnaire. Gowers et al [66] found overall low levels of agreement between the HoNOSCA and the HoNOSCA-SR (a consumer-rated version of the instrument for adolescents) at an individual level, although some groups (e.g., outpatients with eating disorders) were exceptions. Again, these findings are to be expected, given that instruments that rely on information from different classes of informants are likely to demonstrate lower levels of correspondence than those that rely on informants from the same class.

Other studies have assessed the ability of the HoNOSCA to discriminate between groups of consumers based on their clinical and/or treatment profile. Gowers et al [3,4] and Yates et al [64] found that the HoNOSCA could distinguish between consumers in inpatient and outpatient settings and between consumers presenting to clinics with different areas of focus, respectively. Harnett et al [61] found that HoNOSCA total scores were associated with the number of critical incidents in which adolescent consumers were involved. Manderson and McCune [67], Brann et al [63] and Harnett et al [61] found that the HoNOSCA yielded coherent age/sex results – e.g., boys scored higher than girls on Item 1 (Problems with disruptive, antisocial or aggressive behaviour) but lower on Item 9 (Problems with emotional and related symptoms), and younger children scored higher than older children on Item 5 (Problems with scholastic or language skills) but lower on Item 3 (Non-accidental self-injury). Brann et al [63] also reported that the HoNOSCA yielded intuitive results when they considered diagnosis – e.g., consumers with attention deficit and conduct disorders scored highest on Items 1 and 2 (Problems with disruptive, antisocial or aggressive behaviour, and Problems with over-activity, attention or concentration). Similarly, Bilenberg [65] found that high HoNOSCA total scores were associated with comorbidity.

#### Predictive validity

Brann [62] found that HoNOSCA total scores at community assessment could discriminate between adolescents who later received treatment from intensive outreach teams and their counterparts who progressed to other forms of community care.

#### Test-retest reliability

There are few published studies on the test-retest reliability of the HoNOSCA, and those which do exist are arguably studies of the sensitivity to change (or lack of change) of the instrument, since they cover considerable time periods and consider stability in relation to other measures. Garralda et al [68] examined the test-retest reliability of the instrument over a six month period, for consumers for whom clinicians indicated there had been no change on a global rating scale, and reported a figure of 0.69. Similarly, Brann [62] reported correlations of 0.80 over three months and 0.76 over five months when he examined the instrument's test-retest reliability, again in a group of consumers who were judged not to have changed over the given period. Likewise, Harnett et al [61] reported a correlation of 0.80 between initial and subsequent HoNOSCA total scores assessed over a 2–4 week period for inpatient adolescents, whom the authors suggested would be likely to remain relatively stable after a 'settling in' period.

#### Inter-rater reliability

Studies have consistently found that the majority of Section A items demonstrate good or very good inter-rater reliability. However, there is less agreement about which items perform poorly. For example, Brann et al [63] reported a particularly low intra-class correlation (0.06) for Item 10 (Problems with peer relationships), but Gowers et al [3,4] found that this item achieved an intra-class correlation of 0.77.

There is also debate about the inter-rater reliability of Section B. Gowers et al [3,4] found that the two items comprising this section each had good inter-rater reliability: Item 14 (Problems with knowledge or understanding about the nature of the child or adolescent's difficulties) and Item 15 (problems with lack of information about services or management of the child or adolescent's difficulties) had intra-class correlations of 0.73 and 0.78, respectively. By contrast, the equivalent figures in a later study by Garralda et al [69] were 0.27 and 0.03.

#### Sensitivity to change

Three approaches have been taken to assessing the ability of the HoNOSCA to detect change. The first and methodologically weakest approach involves simply determining whether HoNOSCA total scores change over time, with no reference to whether this reflects real change. In the original field work associated with the development of the HoNOS, for example, Gowers et al [3,4] noted that 'the HoNOSCA demonstrated satisfactory sensitivity to change, with a mean overall reduction in total scores of 38% between rating points, on average nearly three months apart'. Manderson and McCune [67] made a similar observation, as did Harnett et al [61].

The second approach examines the correspondence between change as assessed by the HoNOSCA and change as defined by the difference between scores on other measures. Studies by Gowers et al [66], Garralda et al [68] and Bilenberg [65] have reported changes in HoNOSCA total scores that are comparable in direction and magnitude with other clinician-rated measures, such as the Children's Global Assessment Scale and the Global Assessment of Psychosocial Disability, and, to a lesser extent with parent- and/or consumer-rated measures such as the HoNOSCA-SR, the Behaviour Check List and the Strengths and Difficulties Questionnaire.

The third approach uses global outcome judgements as the 'gold standard'. Typically, these require clinicians (or parents/referrers) to indicate whether the consumer has improved, deteriorated or remained stable, via some sort of Likert scale. Studies by Gowers et al [3,4], Garralda et al [68], Brann et al [62,63] and Bilenberg [65] have all reported close correspondence between change (or lack of change) recorded on the HoNOSCA and such global judgements.

#### Feasibility/utility

Studies that have questioned clinicians about the feasibility/utility of the HoNOSCA have generally found them to be positive about its brevity and ease of use, its clinical utility, and its ability to be incorporated into routine practice. Their main concerns have related to the instrument's applicability to children aged under five, its emphasis on child/adolescent symptoms and functioning, and its failure to take into account context. Some clinicians have also questioned whether it may be less useful in the case of particular disorders [3,4,65,67,69].

These and other studies have further considered feasibility/utility by examining the behaviour of services and individual clinicians. For example, Gowers et al [3,4] reported that in the original HoNOSCA field trial none of the sites dropped out and 71% of consumers were rated at both Time 1 and Time 2. They continued to report optimal completion rates in their later work [4].

### HoNOS65+

#### Content validity

During initial HoNOS65+ development, Burns et al [5] asked mental health professionals working with older consumers to review the content of the HoNOS. This process resulted in modifications to the glossary to address their concerns regarding the comprehensiveness of the instrument for older consumers [70]. Since this time, ongoing issues have been noted anecdotally, and further refinements to the glossary have been made [71-73].

#### Construct validity

There is a paucity of evidence on the construct validity of the HoNOS65+. The only relevant data come from the original pilot work by Burns et al [70], where a factor analysis revealed that four factors accounted for 57.4% of the variance in HoNOS65+ item scores.

#### Concurrent validity

Studies by Burns et al [70], Mozley et al [74], Spear et al [75] and Bagley et al [76] have examined the correlations between the HoNOS65+ and more established clinician-rated measures that assess similar domains. Reasonable correlations have been observed between the HoNOS65+ total score and the Mini-Mental State Examination [70,74,75], Crighton Royal Behaviour Rating Scale [70], and Barthel Activities of Daily Living Index [70].

As a general rule, however, stronger correlations have been observed between specific HoNOS65+ items and other instruments:

• Item 4 (Cognitive problems) with the Mini-Mental State Examination [70,75];

• Item 6 (Problems associated with hallucinations and delusions), Item 7 (Problems with depressive symptoms), Item 8 (Other mental and behavioural problems) and Item 9 (Problems with relationships) with the Brief Psychiatric Rating Scale [70];

• Item 4 (Cognitive problems), Item 5 (Physical illness or disability problems) and Item 12 (Problems with occupation and activities) with the Barthel Activities of Daily Living Index [70];

• Item 1 (Behavioural disturbance), Item 4 (Cognitive problems), Item 5 (Physical illness or disability problems), Item 7 (Problems with depressive symptoms), Item 8 (Other mental and behavioural problems), Item 10 (Problems with activities of daily living), Item 11 (Problems with living conditions) and Item 12 (Problems with occupation and activities) with the Crighton Royal Behaviour Rating Scale [70]; and

• Item 1 (Behavioural disturbance), Item 4 (Cognitive problems), Item 9 (Problems with relationships) with the Brief Agitation Rating Scale [75].

There are exceptions, however. Equivocal findings have been reported regarding the relationship between HoNOS65+ Item 7 (Problems with depressive symptoms) and the Geriatric Depression Scale. The original pilot found the correlations between Item 7 and individual items on the Geriatric Depression Scale were good, but that there was no significant correlation between it and the total score [70]. Later studies have produced conflicting results, with one finding a good correlation between Item 7 and the Geriatric Depression Scale [75] and the other finding that the former detected only a minority of the consumers identified as depressed by the latter [76].

A few studies have investigated the ability of the HoNOS65+ to discriminate between different consumer groups. Burns et al [70] found the instrument was able to discriminate between consumers with dementia and those with functional psychiatric disorders, with the former scoring higher on Item 1 (Behavioural disturbance), Item 4 (Cognitive problems) and Item 10 (Problems with activities of daily living), and the latter scoring higher on Item 2 (Non-accidental self injury), Item 7 (Problems with depressive symptoms), Item 8 (Other mental and behavioural problems). Spear et al [75] reported similar findings, demonstrating that consumers with dementia generally had higher HoNOS65+ total scores than those with mood disorders, but had lower scores on the symptoms subscale.

#### Predictive validity

No studies available.

#### Test-retest reliability

No studies available.

#### Inter-rater reliability

Burns et al [70] and Spear et al [75] both found inter-rater reliability to be good to very good for most items. Burns et al found that only Item 2 (Non-accidental self-injury), Item 10 (Problems with activities of daily living), Item 11 (Problems with living conditions) and Item 12 (Problems with occupation and activities) did not consistently perform well. In Spear et al's study, Item 4 (Cognitive problems), Item 5 (Physical illness or disability problems) and Item 9 (Problems with relationships) demonstrated only poor to moderate inter-rater reliability. Allen et al [71], by contrast, found problems with a broader range of items, largely related to difficulties in interpretation.

#### Sensitivity to change

Spear et al [75] found that consumers showed improvement on all HoNOS65+ subscales and on the HoNOS65+ total score between assessment and discharge from inpatient and community services, and that the discharge HoNOS65+ total score and the change in HoNOS65+ total scores showed moderate but significant correlations with the Clinician's Interview Based Impression of Change Scale.

#### Feasibility/utility

In the original pilot, Burns et al [70] assessed the feasibility/utility of the HoNOS65+ by asking raters whether or not they would find the instrument helpful in working with individual consumers; 39% indicated it would be very useful and 50% that it would be of some use. Spear et al [75] reported similar findings. In both studies, almost all respondents reported that it was easy to administer.

Feasibility/utility have also been considered in terms of uptake, both at a national level and at a service level. Reilly et al [77] conducted a survey of old age psychiatrists across the UK, and found that 18% reported that the HoNOS65+ was being used in their service. Spear et al examined the proportion of episodes of care at which the HoNOS65+ was administered within a single service, and found completion rates of 96%.

Other studies have examined the feasibility/utility of the HoNOS65+ more generally, considering issues that have arisen during implementation. Allen et al [71], for example, observed that clinical leadership and timely feedback were crucial, as were minimising the paperwork burden and clarifying analysis and reporting issues. In a similar vein, MacDonald [78] argued that suitable infrastructure must be in place, the data must be managed appropriately, and analysis and reporting should be guided by clinicians' requirements.

## Discussion

Table 2 summarises the review's findings. Mostly, the members of the HoNOS family have adequate or good validity, reliability, sensitivity to change and feasibility/utility. That said, some of the psychometric properties of the instruments are under-investigated and therefore warrant closer examination. There may also be scope for additional work on particular psychometric properties, even where some studies have already been conducted, given that the instruments are being used in the context of routine outcome measurement – e.g., inter-rater reliability (given that a number of raters may be involved in administering measures for the same consumer) and sensitivity to change (given that outcome measurement requires a valid and reliable assessment of improvement, deterioration or stability over time).

**Table 2:** Psychometric properties of the HoNOS family of measures

		**HoNOS**	**HoNOSCA**	**HoNOS65+**
**Validity**	**Content**	Good	Insufficient evidence	Insufficient evidence
	**Construct**	Good	Adequate	Insufficient evidence
	**Concurrent**	Good	Good	Good
	**Predictive**	Good	Adequate	Insufficient evidence

**Reliability**	**Test-retest**	Adequate	Adequate	Insufficient evidence
	**Inter-rater**	Adequate	Adequate	Good

**Sensitivity to change**		Adequate	Good	Adequate

**Feasibility and utility**		Adequate	Adequate	Adequate

One caveat should be considered when interpreting these findings. The majority of studies considered in the review examined the psychometric properties of the original instruments, used as per standard instructions. It must be acknowledged that various modifications have been made to the instruments, to cater for the local context. So, for example, in Australia when the instruments are being used at discharge from an acute inpatient setting, the rating period is the last three days rather than the last two weeks (in recognition of the brevity of such admissions). As yet, no formal psychometric testing has been applied to the modified instruments, and there is a question about the extent to which the findings as they relate to the standard instruments can be generalised.

## Conclusion

This caveat aside, it can be concluded that that, collectively, the HoNOS family of measures can assess outcomes for different groups on a range of mental health-related constructs. Where tested, their psychometric performance is adequate or better. This is important, because it means they can be regarded as appropriate for routinely monitoring consumer outcomes, with a view to improving treatment quality and effectiveness.

## Competing interests

The author(s) declare that they have no competing interests.

## Authors' contributions

JP, PB and TC devised the conceptual framework for the review. JP, PB, PK and MW identified and retrieved all references. JP, PK, SD and MW extracted relevant information from the references, reviewed the measures, and drafted the report upon which the paper is based. All authors contributed to drafting and re-drafting the paper.
